# Challenges for a Maternal-Care Health Recommender System in Indonesia: Formative Preimplementation Qualitative Study

**DOI:** 10.2196/73726

**Published:** 2026-02-06

**Authors:** Rinto Priambodo, Putu Wuri Handayani, Rizal Fathoni Aji, Kaharudin Dimyati

**Affiliations:** 1 Faculty of Computer Science University of Indonesia Depok Indonesia; 2 Department of Informatics Faculty of Technology and Design Universitas Pembangunan Jaya Tangerang Selatan Indonesia; 3 Department of Electrical Engineering Faculty of Engineering University of Malaya Kuala Lumpur Malaysia

**Keywords:** health recommender system, maternal care, antenatal care, public health surveillance, health information systems, preimplementation

## Abstract

**Background:**

Maternal evaluation during routine antenatal care visits may reduce maternal morbidity and mortality by identifying and addressing issues early on. A health recommender system could help health professionals and pregnant women monitor daily health parameters, provide tailored recommendations, and support timely antenatal care.

**Objective:**

This study aims to qualitatively analyze challenges in the preimplementation of health recommender system for maternal care in Indonesia as perceived by multiple stakeholders, including health care providers, patients, health system managers, government officers, and technology vendors.

**Methods:**

The methodology used a qualitative approach, where qualitative data were obtained from interviews of 37 respondents from multiple stakeholders, consisting of 15 health workers and 15 patients from private and government health care facilities, 4 officers from government health offices, 2 directors of health application vendors, and 1 manager from a private health clinic. These semistructured interview results were analyzed using thematic analysis.

**Results:**

This qualitative study identifies key challenges in implementing a health recommender system for maternal care in Indonesia across the people, process, infrastructure, and policy dimensions. Intercoder reliability for the coding process demonstrated almost perfect agreement (Cohen κ=0.90), supporting the consistency of the coding process. Six major challenges were revealed, mostly regarding skill, accuracy, completeness, timeliness, cost, and standardization. These 6 major challenges were mentioned 96 times, accounting for 64.43% of all codes extracted from the interviews. These findings emphasize the value of user involvement in system design to meet health care professionals’ and patients’ needs, technical advancements to foster trust and support effective decision-making, as well as enhanced data accuracy, reliable and timely service delivery, cost management, and clear regulatory standards.

**Conclusions:**

This formative, preimplementation qualitative study highlights the importance of involving users in system design and future implementation to meet the needs of health care professionals and patients. Reducing input errors and improving system reliability are critical to building trust and supporting effective point-of-care decision-making and, in later phases, facility-level monitoring as part of public health surveillance. Adherence to regulatory standards and the establishment of standardized guidelines will be key to enabling broader implementation. Further usability, feasibility, and pilot studies are required before any evaluation of effectiveness.

## Introduction

### Overview

The global maternal mortality ratio is expected to be <70 per 100,000 live births by 2030, according to the Sustainable Development Goals [1]. The World Health Organization (WHO) report on maternal mortality, however, shows that while the rate of maternal deaths has dramatically declined, a sizeable gap needs to be closed before this goal is met, and progress varies among nations [2].

Timely and sufficient antenatal care (ANC) visits are known to reduce maternal mortality [3]. Among the major causes of maternal mortality, hypertensive disorders during pregnancy are the leading cause following postpartum hemorrhage [4,5]. Although a few of these problems might have been present before getting pregnant, most complications happen during pregnancy and can be prevented with routine ANC visits [6]. Maternal evaluations during ANC visits may help identify and address issues early on, reducing risks of morbidity and death for both the mother and the unborn child [7]. Critical illness in pregnancy, like hypertensive disorders, is often diagnosed during pregnancy at prenatal visits, as prenatal care affords the opportunity for prevention, early diagnosis, management, and treatment of hypertensive disorders [8]. Therefore, the WHO released recommendations to ensure an adequate number of ANC contacts, improve continuity of care, promote healthy behaviors, provide dietary supplements, and encourage both community involvement and home visits for ANC [9].

In Indonesia, ANC activities face challenges regarding health facility access and quality, the availability of health workers, and socioeconomic barriers such as cost limitations and education gaps [10]. Maternal mortality remains high in Indonesia, and the reduction in the maternal mortality ratio is among the slowest in Southeast Asia, even though most women attend the recommended number of ANC visits and give birth with a skilled birth attendant [11]. Baharuddin et al [12] also found that the major causes of maternal deaths were preventable, and factors that contribute were commonly health worker–oriented and related to quality of care in hospitals. Indonesia also faces urban-rural disparities in health care services with a mixed public-private delivery system and uneven digital-health uptake [13,14]. Studying this context provides transferable insights for low- and middle-income countries facing similar constraints in digital health adoption.

Using technology to monitor daily health parameters in a normal, everyday environment can help reduce hospitalization and lower medical expenses [15]. Noninvasive sensor technologies can be used to track user behavior, providing insights into how daily lifestyle choices influence health [15]. Additionally, recommender systems in health care have the ability to provide patients and physicians with tools and support, assisting in the ongoing observation and diagnosis of chronic illnesses [15].

ANC activities include not only health education and promotion but also the detection, prevention, and treatment of complications based on measurements taken during regular visits [7]. A health recommender system (HRS) for maternal care could use mobile sensors and portable diagnostic devices to automatically collect these measurements [16-18]. The data required for generating recommendations could be obtained from user inputs, the patient’s health records, and connected medical devices [16,19-29].

Studies by Etemadi et al [30], Croon et al [31], Tran et al [32], and Pincay et al [33] offer insights into the methods and techniques used in HRS and the areas where HRS can be applied. Tran et al [32] explore the types of users who could benefit from different kinds of recommendations provided by HRS and examine user satisfaction with those recommendations. Furthermore, Croon et al [31] and Pincay et al [33] discuss how recommendations should be delivered to users and propose design principles that can be applied to HRS research. While these studies [30-33] address some of the challenges faced by HRS, there is still a gap in the literature regarding a comprehensive exploration of implementation challenges from multiple stakeholder perspectives, such as health care professionals, patients, app developers, and government health officers [30-33]. Existing research predominantly focuses on technical aspects, system evaluation, or specific user groups, like patients or medical experts in general, without sufficiently addressing the broader challenges faced by key stakeholders in maternal care delivery. Additionally, these studies often lack consideration of domain-specific health constructs, such as asthma, pregnancy, and iron deficiency.

### HRS for Maternal Care

An HRS can be defined as a system designed to assist health professionals or patients in decision-making and personalized health care by filtering through large amounts of data and generating meaningful recommendations [15]. These systems could learn user behaviors and adapt their recommendations depending on the domain and the characteristics of available health records [15]. An HRS should be sensitive to patient needs, attitudes, and the specific context of health and disease management to ensure its effectiveness [15]. Such systems accommodate 2 main types of end users: health professionals, who can access additional resources like research articles or clinical guidelines, and patients, who receive high-quality, evidence-based content [15]. Recommendations can be generated based on users’ previous interactions or explicit preferences using methods like collaborative filtering, content-based analysis, and knowledge-based analysis, with many systems integrating multiple techniques and incorporating additional data types, such as time, location, and social information, to optimize performance [32]. According to Saha et al [15], HRS can be categorized into 2 types: one that continuously monitors and diagnoses chronic diseases to support doctors and patients with advice and predictions, and a content-based system that helps users explore conditions related to their illness through semantic analysis. While most HRS use graphical user interfaces, some distribute information via mobile apps or web-based mobile interfaces [17,23-25,27,34,35]. Alternatively, other systems present recommendations through web interfaces [31].

The WHO recommendation for pregnancy care includes regular checkups and routine screenings to maintain wellness, detect complications early, and prevent conditions that complicate delivery [9]. The HRS aids in diagnosing and monitoring patients over time by using personal health records gathered from measurements taken by health care providers or the patients themselves [15]. Using HRS for maternal care, pregnant individuals can receive recommendations on diet, exercise, medication, or consultations [16,18,23,24,28]. These systems may incorporate portable diagnostic tools and mobile sensors to enable automatic measurements [16,18,26]. Data from connected medical devices, along with user input in patient health records, can be used to provide recommendations that assist with diagnosis and monitoring during ANC [16,19-29]. While previous studies highlight the potential of HRS to monitor pregnancies and mitigate risks, challenges in implementation, particularly from physicians’ perspectives, have also been explored [36].

### Conceptual Model of Research

SERVQUAL (Service Quality) is one of the widely used and accepted methods for service quality measurement [37]. The quality of health care service, as assessed by the SERVQUAL model, can be determined by examining the gap between desirable and current conditions based on 5 dimensions: tangibles, reliability, responsiveness, assurance, and empathy [38]. Furthermore, the HEALTHQUAL (Healthcare Service Quality) model expands these dimensions by including health care–specific aspects like safety, efficiency, and patient outcomes [39]. Additionally, Nemati et al [40] highlight availability, affordability, and caring as essential factors to consider in assessing health care quality. Together, these elements support a comprehensive approach to measuring care quality from the perspective of both patients and health workers [39].

In analyzing the challenges of implementing HRS in Indonesia, the framework from Handayani et al [41] also provides valuable insights. The study by Handayani et al [41] on strategic hospital services quality in Indonesia identifies key dimensions necessary for improving health care services, including human resources, processes, policy, and infrastructure. Each of these dimensions plays a crucial role in the successful implementation of health care systems, including HRS for maternal care. In the analysis, we used the model to orient coding while allowing inductive themes to emerge.

The people or human resources dimension refers to the quality and professionalism of health care staff, emphasizing empathy, communication, and skills, which are critical in building trust and patient satisfaction [41]. Health care professionals are expected to possess the necessary technical skills, medical knowledge, and expertise, while also being empathetic, compassionate, and trustworthy [42]. Empathy in health care is reflected in the ability of medical staff to understand and acknowledge the patient’s situation during treatment, demonstrating personal concern for each individual [39]. It is further expressed through prioritizing patient needs, offering personalized care, showing courtesy to both patients and their families, and providing emotional support [37].

The process dimension focuses on the responsiveness and reliability of service delivery, including timely, accurate, and efficient health care operations [41]. Reliability refers to the ability to deliver consistent, dependable services, characterized by timely health care, transparent processes, and standardized treatments and procedures [37]. Responsiveness involves having quick, efficient, and customer-focused systems, such as an effective triage system for rapid emergency response and clear communication [37]. Efficiency in health care quality relates to the extent to which processes and operations are optimized to deliver effective services [39].

The policy dimension in health care involves establishing clear guidelines and standards that govern practices and ensure compliance with regulations, providing assurance to both patients and staff [41]. A significant challenge in this context is the cost-effective implementation of proven clinical interventions, with common barriers such as limited time and staffing resources [43]. Safety quality aspects further emphasize maintaining high staff qualifications, fostering confidence in service delivery, and ensuring a safe environment for both patients and employees [39].

Lastly, the infrastructure dimension pertains to the availability and quality of physical and technological resources, such as medical equipment and hospital facilities, which support the delivery of high-quality care [41]. Advanced medical equipment and technology are central to tangible quality aspects, ensuring high standards in health care alongside the best medical staff [39]. The equipment used in health care services also defines tangibles, referring to both the physical facilities and the condition of the tools used in delivering care [37].

### Objective

In this study, an HRS refers to patient-facing and clinician-supporting features embedded in routine ANC workflows that generate patient-specific, actionable suggestions for pregnant women and/or clinicians, using guideline-based and/or machine learning–driven approaches. We exclude general health information systems and stand-alone prediction tools that do not produce actionable recommendations. Thus, the aim of this study was to identify implementation challenges of maternal-care HRS in Indonesia from the perspectives of multiple stakeholders. Accordingly, we addressed the following research question: What are the key challenges in implementing a maternal-care HRS in Indonesia, as perceived by multiple stakeholders? The findings of this study are expected to inform stakeholder-aligned design and implementation strategies to support adoption within routine ANC workflows.

## Methods

### Study Design

This formative, preimplementation qualitative study used a purposive sampling method to select respondents and elicit stakeholder perspectives on anticipated barriers, enablers, and surveillance-relevant requirements for future implementation; no HRS was deployed or tested in practice. This study followed the COREQ (Consolidated Criteria for Reporting Qualitative Research) guidelines as a comprehensive checklist to ensure complete reporting of key components of qualitative research (Multimedia Appendix 1) [44]. We used purposive, iterative sampling with concurrent analysis. Recruitment proceeded interview-by-interview and ceased when additional interviews yielded no substantively new codes (thematic saturation). A total of 37 participants from 32 organizations were interviewed. Among the respondents were 12 medical doctors, who were all specialists in obstetrics and gynecology, and 3 midwives. Those medical doctors and midwives worked in public hospitals (4 participants), private hospitals (11 participants), and private clinics (4 participants). It is important to note that in Indonesia, a medical doctor can work in more than one health care facility. Additionally, we interviewed 15 pregnant women who were patients at private clinics (12 participants) and community health care centers (3 participants). Four participants from government agencies represented regulatory bodies, while 2 participants with IT backgrounds, holding director positions at health application development companies, provided insights into the technological aspects. The remaining participant was part of the management team of a private mother and child health clinic.

In terms of demographics, the group consisted of 31 females and 6 males. Most participants lived or worked in Banten province (29 participants) in a region in the greater Jakarta area, characterized by urban infrastructure with well-developed health, communication, and transportation systems. The remaining participants worked in Jakarta province (6 participants) and West Java province (2 participants), which are also near the greater Jakarta area. To complement this urban perspective, we additionally interviewed representatives from government offices, such as the Ministry of Health and public health offices, whose system-level viewpoints provided a counterbalance to facility-level experiences. Table 1 shows the summary of the participant characteristics.

**Table 1:** Participant characteristics in a formative, preimplementation qualitative study of a maternal health recommender system in Indonesia (n=37).

Characteristics of participants			Participants, n
**Respondent group**			
	Regulator	4	
	Specialist	12	
	Midwife	3	
	Health app developer	2	
	Clinic management	1	
	Patient	15	
**Sex**			
	Male	6	
	Female	31	
**Province**			
	Banten	29	
	Jakarta	6	
	Jawa Barat	2	
**Type of organization**			
	Ministry of Health (DTO^a^)	1	
	Health office	2	
	Community health center (Puskesmas)	4	
	Government hospital	4	
	Private hospitals	11	
	Private clinic	4	
	Professional associations	4	
	Health app vendors	2	

^a^DTO: Digital Transformation Office.

### Ethical Considerations

Ethical approval for this study was obtained from the Faculty of Computer Science at the University of Indonesia (approval number S-15/UN2.F11.D1.5/PPM.00.00/2024). Additional approval for data collection in Tangerang Selatan was provided by the local government health office (letter number 400.14.5.4/1560/SDK). All participants received information about the study purpose, procedures, voluntary nature of participation, and their right to withdraw at any time. Written or verbal consent was obtained before each interview. To ensure confidentiality, transcripts were anonymized and deidentified prior to analysis, with access restricted to the research team. Participants received no financial compensation or transportation allowance. No identifiable images or personal information are included in this manuscript or supplementary materials; all quotations are anonymized.

### Data Collection

Semistructured interviews were used to gather data between August 23, 2024, and November 13, 2024. The interview was conducted via phone call, Zoom meeting (Zoom Communications), or face-to-face dialogue following the availability or the preference of each participant. Each interview lasted between 30 and 60 minutes and was recorded after acquiring the participant’s verbal consent, witnessed by the interviewer. The recordings were transcribed, then translated, and the resulting data were anonymized. The interview questions were created to investigate the preimplementation of an HRS for maternal care, focusing on the dimensions of people, processes, infrastructure, and policy to ensure a comprehensive understanding of the challenges and opportunities (the interview questions are available in Multimedia Appendix 2).

### Data Analysis

**Figure 1:**
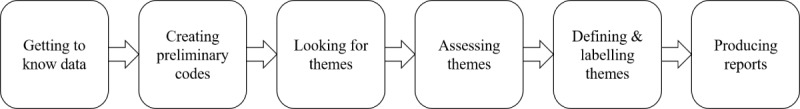
Data analysis workflow for a formative, preimplementation qualitative study of a maternal health recommender system. Semistructured interview transcripts (n=37; regulators, clinicians, midwives, patients, app vendors, clinic manager) were analyzed in QDA Miner Lite using a 6-step thematic analysis (familiarization, initial coding, searching themes, reviewing themes, defining/naming themes, and reporting).

To examine different perspectives from research participants, thematic analysis was used to analyze interview data in QDA Miner Lite (Provalis Research). The 6 steps of a thematic analysis as described in Harahap et al [45] are as follows: getting to know the data, creating preliminary codes, looking for themes, assessing themes, defining and labeling themes, and producing reports (Figure 1).

We first read the interview transcripts several times to become acquainted with the meanings of the data in order to become familiar with them. Every transcript file has a name that corresponds to the case and the original creation date. Second, we used QDA Miner Lite to arrange the dataset before creating the programs. All of the first codes that were discovered were noted, along with short notes and quote samples. Third, we categorized codes into subthemes and then categorized the subthemes into themes inductively. The research framework was used to help search for themes and generate initial themes from codes. When appropriate, the research framework deductively guided the organization of candidate themes without forcing fit, and we iteratively refined the structure to maintain coherence with the data. Subthemes were then derived from the coded data and represented as child nodes. In QDA Miner Lite, the themes were shown as parent nodes, and the codes and subthemes were shown as child nodes. A codebook was used to maintain the evolution and categorization of codes and subthemes. To support coding rigor, one researcher initially developed the preliminary codes and subthemes, which were subsequently reviewed by another researcher. The 2 researchers then discussed and refined the coding framework until agreement was reached on code definitions and theme boundaries. To quantitatively assess coding consistency, intercoder reliability was calculated using Cohen κ. This hybrid approach, where collaborative validation was combined with quantitative agreement testing, ensured interpretive consistency, minimized bias in theme development, and provided transparency regarding coding reliability in this formative qualitative study [46]. To limit confirmatory bias, we organized themes without forcing fit, retained nonaligned inductive codes as emergent/cross-cutting, held consensus meetings to reconcile coding differences, used constant comparison across transcripts, and actively looked for negative/variant cases to revise themes or mark their boundary conditions. Fourth, we evaluated the transcripts to make sure the chosen quotes or coded data fit the subtheme and went over the codes for each subtheme to assess their relevance. Subthemes were then examined alongside themes by the authors. Examining and contrasting earlier research was another step that complemented the process of connecting themes, subthemes, and codes. Fifth, we verified the themes and classification after writing in-depth analyses for each theme in order to refine, define, and name them. Finally, we created the report’s Discussion section, which compares the findings with earlier research that supports our interpretation, and presents the report’s themes, subthemes, and selected quotes. Although we calculated the frequency of each code to indicate the relative prominence of themes across stakeholder groups, these counts were interpreted descriptively rather than statistically. We analyzed the qualitative data until we did not find other themes or reached data saturation. The purpose of reporting frequencies was to illustrate thematic salience and identify the most commonly discussed issues, not to imply quantitative significance or apply statistical normalization.

## Results

### Overview

**Figure 2:**
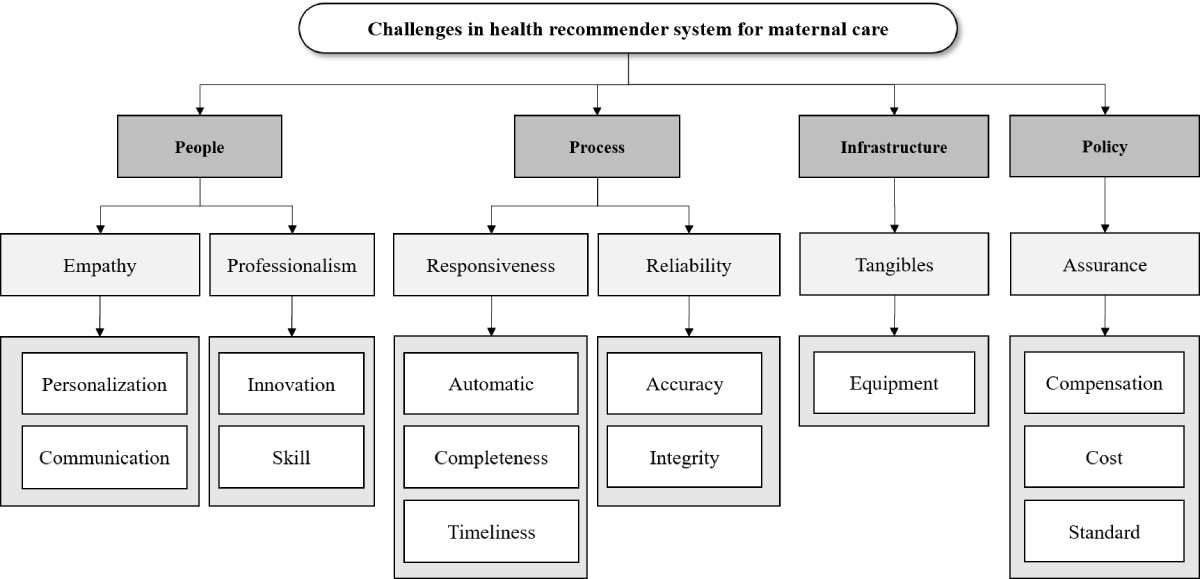
Thematic map of challenges identified from 37 stakeholder interviews on a maternal health recommender system.

We discovered 13 challenges that were represented by codes, where each code was mapped to 6 subthemes and 4 themes thereafter. People, process, infrastructure, and policy are the 4 dimensions reflecting themes. The people dimension has empathy and professionalism as subthemes and 4 codes under each subtheme. Process dimension has responsiveness and with 3 codes and reliability with 2 codes underneath. The infrastructure dimension only has tangible as a subtheme with only 1 code. Lastly, the policy dimension has assurance as a subtheme and 3 codes. These relationships are illustrated in a thematic map in Figure 2.

**Figure 3:**
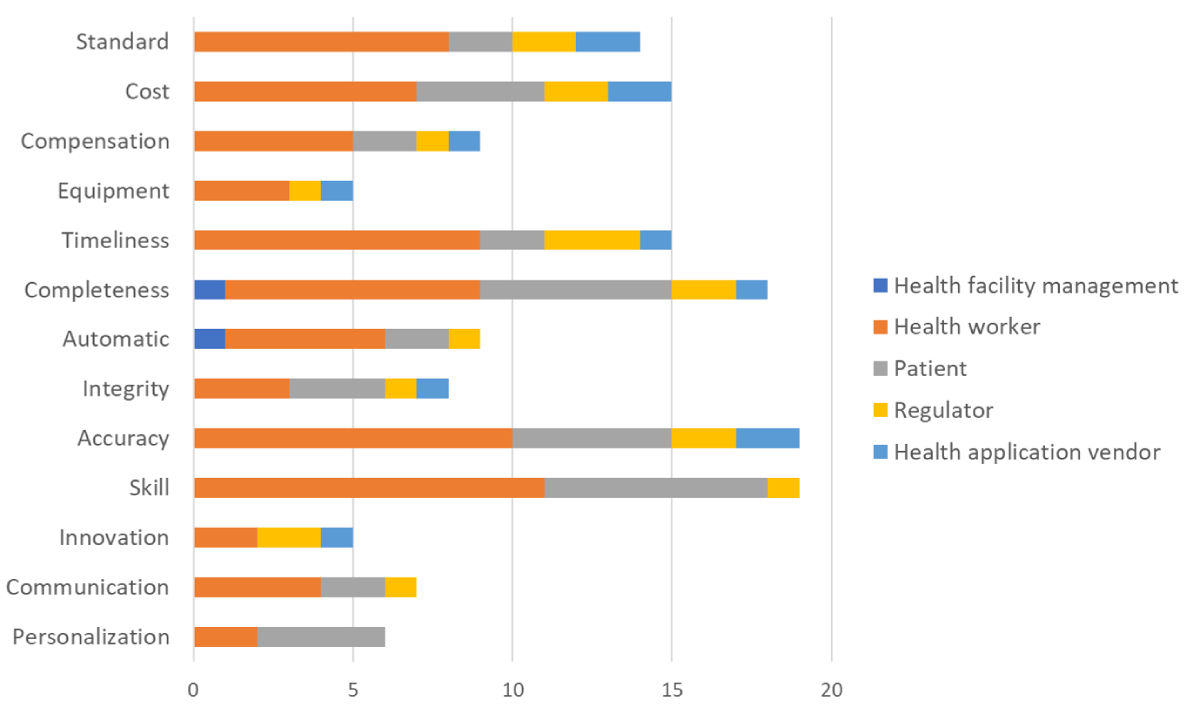
Distribution of code frequencies across stakeholder groups for preimplementation challenges of a maternal health recommender system (n=37 interviews). Higher bars indicate more frequent mentions. Consistent with Table 2, the 6 most-cited challenges (skill, accuracy, completeness, timeliness, standards/standardization, reliability) account for 96 references (64.43% of all coded instances).

The frequency of occurrences of the codes in the transcripts was calculated as shown in Table 2 and illustrated in Figure 3. These frequencies are presented to demonstrate which themes were most salient across participants; however, they should be interpreted descriptively. The counts reflect emphasis in participant discussions rather than normalized or statistically analyzed values. Intercoder reliability for the coded subset demonstrated almost perfect agreement between the 2 researchers, with Cohen κ=0.90, indicating high consistency in the application of the coding framework. Notably, professionalism, particularly the skill subtheme, is emphasized by health workers (12 occurrences) and patients (8 occurrences), indicating the importance of competence in using the system. Similarly, accuracy under reliability is a significant concern for health facility management and patients, highlighting the need for precise system outputs. The completeness of information in responsiveness is a critical issue for health workers and patients, underscoring the need for comprehensive data in the system. Additionally, financial concerns such as cost and compensation are primarily noted by health facility management and regulators, reflecting broader concerns about the system’s affordability and sustainability. Timeliness is also crucial, particularly for health facility management, who emphasize the importance of efficient service delivery.

**Table 2:** Frequency of qualitative codes by stakeholder group in a formative, preimplementation study of a maternal health recommender system (n=37 interviews).

Theme, subtheme, and code							Health facility management (n=1), n		Health worker (n=15), n		Patient (n=15), n		Regulator (n=4), n		Health application vendor (n=2), n		Total, n
**People**																	
	**Empathy**																
		Personalization					0	2		4		0		0		6	
		Communication					0	4		2		1		0		7	
	**Professionalism**																
			Innovation				0	2		0		2		1		5	
			Skill				0	11		7		1		0		19	
**Process**																	
	**Reliability**																
				Accuracy			0	10		5		2		2		19	
				Integrity			0	3		3		1		1		8	
	**Responsiveness**																
					Automatic		1	5		2		1		0		9	
					Completeness		1	8		6		2		1		18	
					Timeliness		0	9		2		3		1		15	
**Infrastructure**																	
	**Tangible**																
					Equipment		0	3		0		1		1		5	
**Policy**																	
	**Assurance**																
						Compensation	0	5		2		1		1		9	
						Cost	0	7		4		2		2		15	
						Standard	0	8		2		2		2		14	

### People

The people dimension covers the needs and challenges of both health care providers and patients. Through qualitative analysis of interviews, several key themes emerged that reflect the human factors influencing the system’s adoption and functionality. These themes include empathy and professionalism concerns, illustrating the importance of tailored information, interactive communication, and the delivery of new technology, as well as individual factors.

#### Empathy

##### Personalization (Need for Individualized and Specific Maternal Information)

General apps rarely meet specific maternal needs, offering only basic advice and leaving concerns unresolved. The current maternal health book offers very basic advice, such as directing users to see a doctor in case of danger signs, but it lacks detailed recommendations. A more personalized solution (eg, app or chatbot) could guide symptom causes and provide tailored recommendations.

Not everyone has the same problem. An application is for, for the general population, it’s like saying, actually it’s for the general population, right, and everyone has a different individual function.

DR1

##### Communication (Interactive Communication)

Patients need direct interaction with doctors to build trust and gain clarity, but constant personal contact can overwhelm doctors. A potential solution is an app that schedules and manages communication, ensuring patients get guidance without overburdening doctors.

They love it when they can have direct contact with us. On the other hand, when we give out our contact information, it can sometimes be disruptive.

DR3

Some patients feel a maternal HRS is unnecessary because they already have easy access to health workers and facilities. For those near health centers, face-to-face education is faster and more convenient than using an app, and comprehensive apps are hard to find; direct access to doctors is often enough.

Because the neighborhood is close, for their education, it’s faster to came here. They are also more focused, right. That’s why it may not be really necessary to have an application.

GO3

#### Professionalism

##### Innovation (Time and Effort in New Tech Delivery)

Many developers struggle to reach product-market fit because solutions do not align with clinicians’ workflows or pregnant women’s needs, leading to low interest.

Maybe there is something that we still miss ... As long s we haven’t found a product market fit or product solution fit, this can’t continue more massively.

VD2

Adoption also slows due to the need to train users (providers and patients). During transition, old systems run in parallel until the new one embeds in daily work, and technological/infrastructure gaps add friction.

The Satu Sehat platform [Indonesia’s integrated health data platform], the main goal is actually initially reducing such applications. But during this transition period, they are still input in their respective applications.

GO2

##### Skill (Individual Factor)

Applications in health care should be designed to be easy to use, with information that is simple to understand, provided to both health care providers and patients. Some users may find traditional methods, like writing in a book, more straightforward, leading to more complete and detailed notes. However, others prefer the convenience of digital tools, finding it easier to access information through gadgets or by searching online. The system must also consider user preferences for different types of interaction with the application, ensuring that it is intuitive and adaptable to individual needs. Moreover, patients should be equipped with the necessary knowledge to accurately measure health indicators at home and correctly interpret the results.

Sometimes there are patients who don’t understand, but it turns out that it is already written by the doctor, so it is more, I rely more of a book than that, for example, sometimes it is also more complicated to work than to write, so sometimes it is even more complete what is written, which is in a book than what is typed.

DR2

### Process

The process dimension addresses issues related to reliability and responsiveness. The challenges identified within this dimension include issues related to the accuracy of data input, quality of output, and data integrity. Additionally, there are concerns about the need for automated processes, the lack of data integration, and incomplete information provided to users. Timeliness is another key concern, particularly in terms of ensuring continuity of service, delivery time of service, and improving time efficiency in patient handling.

#### Reliability

##### Accuracy

###### Incorrect Input

Reliability in the implementation of a health care information system is dependent on the accuracy of the data input. Manual data entry, while sometimes necessary, is prone to human error, leading to inaccuracies that can compromise patient care. To mitigate these issues, an application could use a guided form within the system that can help standardize input and reduce the risk of mistakes. Otherwise, as some users point out, the process to avoid inaccuracies requires careful checking and rechecking, and if errors are found, it may require redoing the entire examination, which is time-consuming and frustrating.

I think there’s always some manual input involved, like entering lab results, which is probably done manually. Because the lab system isn’t connected to the Satu Sehat, so the lab staff have to enter it manually. Hopefully, there won’t be any human error during the entry.

DR7

Technical issues within the application could lead to input errors. Such errors can result in data that are either inaccurately recorded or, in some cases, completely unreadable. This not only disrupts the continuity of care but also poses significant risks to patient safety, as health care providers may be unable to access crucial information when needed. These technical glitches undermine trust in the system, making it imperative to address them proactively.

The problem is that the delivery of the results doesn’t come in even though it’s been sent. It seems like we have updated the patients as of today, right, but we can’t read it later.

GO4

For the system to function effectively, diagnostic devices must be routinely calibrated to ensure they deliver precise measurements. Without regular calibration, there is a risk of inaccurate readings, which could lead to incorrect diagnoses or treatment plans. Additionally, there is a valid concern regarding the sensitivity of diagnostic devices; if a device is not sensitive enough, it may fail to detect subtle but crucial changes in a patient’s condition, further compromising the accuracy of the information within the system.

The better the device, the more sensitive the tool is, right. I’m afraid that for example, like a sports watch, if the sensitivity for the pulse track will also be different, so it will be different from the sensitivity.

DR2

###### Quality of Output

The accuracy of a health care information system for maternal care is not only determined by the correct input and processing of data but also by ensuring that the data are delivered to the right person. If the information reaches the wrong individual, it could lead to significant errors in patient care, including incorrect diagnoses, inappropriate treatments, or delays in critical interventions.

Additionally, another significant challenge is the potential for misinterpretation or the generation of inaccurate recommendations, which can occur if the system’s data are not correctly analyzed or presented. For instance, if the system displays inaccurate information, it can lead to misinterpretation by health care providers or patients, resulting in misguided decisions or actions. Furthermore, discrepancies in critical calculations, such as due date estimates, can cause confusion and erode trust in the system, especially when the simulations provided are significantly off-target.

Last time the simulation was very far from the ... what ... from menstrual tracking. Also when entered into the application. The estimated due date is like a month different.

PS10

There is often an assumption that health apps may not provide all the necessary information about a patient’s condition, leading to a lack of motivation to use them. Some patients may not be aware of the importance of monitoring their health, or they may lack support from those around them to do so. Moreover, health apps are frequently deprioritized in favor of entertainment or social media unless their use is mandatory. For patients experiencing a second pregnancy, there may be a perception that they already know everything they need to, making them less inclined to use the app.

Even if we make an application, if from the mother herself, her desire to monitor herself is lacking, it is still less useful.

DR8

###### Partial Availability of Required Data

For the system to provide precise and reliable guidance, it must have access to all necessary data inputs, which typically require contributions from both the patient and the physician. It is good that patients understand what data should be available in the system, but examination results from physicians are needed to create recommendations. If any required data are missing or incomplete, the algorithm within the system cannot produce an accurate recommendation, leading to potentially flawed advice that could impact patient care. Therefore, it is important to ensure that both patients and health care providers understand their roles in data input to maintain the system’s accuracy and overall effectiveness.

If the results are patient-based, only based on what the patient knows or experience, maybe the recommendations will not be very accurate.

DR6

##### Integrity (Data Integrity and System Reliability)

Clear accountability for data entry/management builds trust. Users need assurance that data are accurate and protected end-to-end, with strong technical and operational security and not just policies on paper. Many still fear cloud risks.

What is clear to me is a safety issue. Data safety, right. That the cloud is not safe. Yes, I think so. Because this cloud has data everywhere. If someone hacks like that, it’s really troublesome.

DR1

Reliability issues such as crashes and poor connectivity, especially in weak-network areas, undermine confidence and make users hesitate to rely on the system.

Some people are still in trouble if the network is not very strong.

GO4

#### Responsiveness

##### Automatic

###### Need of Automated Process

Linked data can trigger reminders and follow-ups automatically, improving responsiveness for providers and patients. But overcomplex automation is hard to build and maintain and can cause delays/errors.

It’s a bit difficult if it’s a bit complex, if it’s simple, it’s already automatic in our hospital.

DR5

###### Repetitive Action by User

Users often reenter the same data across apps. Interoperability with medical records would allow automatic data pull, reducing manual entry and frustration.

We write everything manually, so we can’t directly pull the data …. If it was already entered into the system, the data could be pulled right away. So, if you want to connect that, that’s how it should work.

DR2

##### Completeness

###### Lack of Data Integration

Users, both patients and health care providers, express a strong preference for an all-in-one solution that consolidates various functions and data sources. Patients, for example, desire the ability to view ultrasound results and track fetal development directly within their app, without having to switch between multiple platforms. The absence of integration between different applications often leads to inefficiencies and complications, making it difficult to manage health care processes smoothly. When systems are not interconnected, simple tasks like managing schedules, setting reminders, or sharing data become cumbersome, requiring multiple actions that could be streamlined through a unified system.

Yes, if you can have one application that covers everything. So there is no need to double record, and there is no need to repeat it.

BD2

When an app is fully integrated with medical records, it can automatically generate reminders and recommendations that are personalized and timely, directly benefiting patient care. However, a common challenge arises when health facility apps are not connected to other applications and are used merely for data collection. This lack of integration limits the system’s ability to provide actionable insights, as it cannot leverage the full spectrum of patient information.

If there could be an app that acts like a personal doctor, reminding you, “Hey, your weight has reached this point,” that would be great. But the issue is that it’s not integrated with medical records.

DR1

###### Partial Information

When only partial information is accessible, it limits the effectiveness of both patient care and health care management. For instance, teleconsultation has inherent limitations because it cannot perform physical examinations, which are vital for accurate diagnosis and treatment. Additionally, patients often find themselves with access to only a portion of their medical information, which can hinder their understanding and engagement in their own care. On the other side, doctors may struggle to obtain comprehensive data on their patients, particularly regarding treatments received elsewhere, leading to gaps in care continuity. Moreover, relying solely on traditional methods, such as paper records, fails to provide a clear view of a patient’s medical history and risk factors, which are essential for informed decision-making.

They don’t see the previous pattern, so they only look at the examination at that time. Meanwhile, when we combine it with some historical data, or indeed the use of this application has been carried out from the beginning of pregnancy, there will be patterns of what risk factors the mother has.

VD1

Another key aspect in the completeness of information is the ease of data recording that digital applications provide. Unlike physical books, which can be easily lost, forgotten, or suffer from scattered and unreadable notes, apps offer a secure and organized way to store important health data. This digital approach reduces the risk of losing critical information and ensures that records are always accessible when needed. However, despite this convenience, it is important to recognize that patients may still struggle to interpret their own health data accurately. Even with easy access to stored information, they often rely on a doctor’s expertise to draw conclusions and make informed decisions about their care.

Yes, I mean, reading is fine, but I don’t want to make assumptions on my own. Sure, for example, with hypertension, you’re supposed to do this and that. That’s the theory. In practice, we need to ask the doctor directly. We can’t conclude on our own. Because it is still, even if you want to take action, you must have the doctor’s approval. Like in my case, I have gestational diabetes, that’s a warning, I can’t decide on my own what to do. I’d be clueless.

PS15

##### Timeliness

###### Continuity of Maternal Care

Health workers need the flexibility to input data into the system while on the move, allowing patients to immediately view results and follow up on their care. This seamless integration is vital for maintaining a continuous workflow. However, the experience is often disrupted when the application crashes, is inaccessible, or lags, especially during critical moments like home visits when access to data is urgently needed.

It would be better if there was already a system that was used when they served while go around, they immediately input at the system and allows the patient to see the results and later in the future so they will be better at following up.

GO1

A maternal care system must not only detect and diagnose problems in a timely manner but also ensure that appropriate follow-up actions are seamlessly integrated into the care process. This means that the system should be designed to automatically prompt and schedule follow-up appointments, reminders, and any necessary additional tests or treatments, ensuring that patients receive continuous care without gaps.

So, with any health-related app, the difficult part is the follow-up. Because in my opinion, these online apps are helpful for initial emergencies, but after that, there’s no follow-up. You can’t just contact the same number or even the same doctor again. It’s unlikely that you’ll be connected to the same doctor again after using the app the first time.

DR5

###### Delivery Time of Service in Maternal Care

Physicians need to take action at the right moment to ensure effective treatment, and early detection of health issues is essential for preventing complications. To support this, the system must be capable of delivering necessary services and information to the right stakeholders exactly when needed. For instance, using instant messages sent directly from the system can significantly enhance awareness and prompt action, especially since stakeholders may not be checking the app regularly.

If they have to open the application every time, there’s a concern that they might miss it. And we also don’t just remind in emails, but we also make interactions in emails.

VD1

###### Time Efficiency in Patient Handling

In a setting where the number of patients is high and time is limited, improving time efficiency becomes essential. A health care information system that optimizes time management allows health care providers to handle more patients effectively without compromising the quality of care. By streamlining processes, reducing unnecessary delays, and automating routine tasks, the system can free up valuable time for health care professionals, enabling them to focus on direct patient care.

There are many pregnant women as patients, as you can see right now, there are 30. And my time is limited, right, so for one patient we need fast.

DR10

### Infrastructure

#### Overview

The infrastructure dimension highlights the tangible challenges related to equipment availability/performance and access to technical support. Key challenges within this dimension were identified, including the availability of required equipment and infrastructure, as well as the difficulty in accessing technical support. For instance, there is a significant gap in network quality and availability between different areas, hindering the system’s effectiveness; in such settings, offline capture-and-sync strategies are used to ensure data transmission. Additionally, the lack of easy-to-reach technical support further complicates the smooth operation and maintenance of the system. Where connectivity issues affect the timing of care or reminders, we discuss implications under process timeliness.

#### Tangible (Equipment)

##### Availability of Required Equipment and Infrastructure

In many cases, the necessary equipment and infrastructure are not uniformly available, leading to significant disparities in health care delivery. The gap between urban centers and suburban or rural areas is often substantial, even within the same region. While downtown areas might have advanced technology and reliable network connectivity, many suburban and rural regions face challenges such as poor network access and inadequate infrastructure. These communication access issues are particularly problematic in health care, where a strong and consistent network is essential for the timely transmission of data, remote consultations, and other critical services.

Yes, that’s why I said because this technology has the obstacle, right, not all regions have a good network, right, that’s why there is offline data transmission. When it is downloaded, it enters like that, so there are various strategies.

DR4

##### Lack of Technical Support

A lack of accessible and timely technical support can lead to frustration, decreased trust in the system, and ultimately, reduced usage. An app provided by the government nationwide could have centralized support that can be difficult to reach. This is especially critical in health care, where delays or difficulties in accessing information can have serious implications for patient care.

No, we usually can’t directly contact the technical support, because that’s the program from the Health Office.

BD2

### Policy

#### Overview

The policy dimension covers the guidelines, regulations, and resources that shape how such systems are developed and applied. Several challenges were identified within this dimension, particularly concerning compensation, cost, and standardization. Key challenges include the lack of alternatives for patient monitoring, budget limitations, and the disparity between costs and perceived benefits. Additionally, the need for standardized application development and strict regulatory compliance is emphasized to ensure that health facilities and developers follow government regulations and medical practice standards. Budget limitations pose a significant challenge, particularly for health facilities with restricted resources, which may hinder the system’s full implementation. Additionally, the balance between cost and benefit is a concern, with potential cost burdens on patients that could diminish the system’s perceived value.

#### Assurance

##### Compensation (Absence of Alternatives for Patient Monitoring)

Clinicians still keep paper records because digital systems are error-prone or go down; paper becomes the source of truth, and outages force providers to reconfirm info with patients. Without a manual backup, progress cannot be tracked during downtime.

They have manual records ... So they have a record if they are going to get around, they have data on which one they want to visit.

GO3

A fragmented app ecosystem also persists: facilities build their own tools due to limited guidance, leading to poor integration. What is needed is a unified, interoperable platform rather than many siloed apps; government support often stops at web resources.

If we’re talking on a national level, why should every health care facility have to create its own app, right? Ideally, they should just be able to integrate.

DR12

##### Cost

###### Budget Limitation in App Implementation

While large hospitals can make significant investments in IT infrastructure, the cost of developing and maintaining health care apps can be prohibitively expensive for smaller institutions. These primary and regional facilities often operate on limited budgets, and local governments, which are responsible for funding these centers, also face financial constraints. The Ministry of Health may not allocate sufficient funding for health care IT, leaving these institutions with inadequate resources to implement and sustain these systems. As a result, even if an app is developed, its long-term viability is at risk if funding stops.

I have also made an application for pregnant women ... but the cooperation was only 3 years, and now it can no longer be accessed.

GO1

While there may be abundant ideas for innovative solutions, the in-house IT teams are often focused solely on managing daily operations and maintaining existing infrastructure, leaving no capacity for the development of new applications. This creates a significant gap, as organizations must then rely on third-party developers to build and implement the system. However, contracting with external developers can be costly, especially for smaller health facilities with limited budgets. This reliance on third-party services further strains financial resources, making it difficult to invest in the necessary IT infrastructure.

The logic in the application can be used, for example, to make recommendations and others whose data has been entered but we’re still limited in resources that the person who works on it also does not available.

GO2

###### Inequal Benefit Compared to Cost

For many patients, the cost burden of using health care technology can be a significant barrier. The technology must be affordable, accessible, and easy to use to encourage widespread adoption. If an app requires payment, subscription fees, or is cluttered with advertisements, patients are often reluctant to engage with it, preferring alternatives that are free or less intrusive.

Number one, of course, the most important thing later is that this technology must be cheap, right? It has to be cheap, anyone can buy it, right?

DR4

Many stakeholders may feel that the high costs associated with developing, deploying, and maintaining such a system are not justified by the benefits it provides. This sentiment is particularly strong when the perceived needs are not fully met by the system, leading to dissatisfaction and reluctance to invest. Additionally, the recent release of a government-backed app can worsen these concerns, as users may question the necessity of investing in a separate system when a potentially more cost-effective or widely supported alternative is available.

It seems that the price is also not bad. Then the hospital may also feel that it is not too necessary because there is no interest either. Maybe it would be very useful if the model was in remote areas like that might be more useful.

DR3

##### Standard

###### Standardization in Application Development

Currently, patients who visit multiple facilities often encounter a variety of record-keeping formats, leading to confusion and difficulty in managing their medical history. This lack of uniformity can be particularly problematic when there are many parties involved in a patient’s care, raising questions about who is responsible for data input and how the information should be managed. Moreover, a standard for connecting medical devices is needed, as each device may operate under different protocols.

So it’s tricky and sometimes because of the custom protocol, they have updates, in the previous backend so that the integration of the device needs efforts and there is no standard.

VD2

###### Regulatory Compliance

Health care providers are required to adhere to strict data privacy regulations, reflecting widespread concerns among patients and providers about the safety and confidentiality of medical data. According to regulations, medical data must remain confidential. However, there is a particular fear that connecting an app to an electronic medical record system could expose sensitive data to risks such as hacking, loss, or tampering. This skepticism is often heightened by concerns over user negligence, as users frequently fail to keep their credentials secure, making them vulnerable to data leaks. In other cases, storing patient data within an application is not allowed by law, yet practical challenges, such as signal problems, can delay the immediate transmission of data, complicating compliance.

We refer to the old law, the old health law, so applications like this are very, very comply. So there is no data at all that can be stored in the application, on a cell phone. Because this medical data is sensitive, and there is a legal impact, if we refer to the old law, all the data must not be stored in ... we couldn’t save in ... save first, in the cache or here in the application or in the cell phone, all data is sent immediately.

VD1

The WHO provides standards and guidelines for medical practice; yet, not all applications easily comply, even with these guidelines in place. Hospitals often face lengthy bureaucratic processes that can hinder the implementation of such systems, while clinics may exhibit more flexibility. Despite the push for connectivity and standardization, there are still a few implementations that successfully send data to a unified platform, and not all vendors possess the capability to ensure seamless connectivity.

So the behavior is somewhat different between hospitals and clinics. We used to fit into the hospital and it is true that they are difficult to change, the bureaucracy is long, one feature can take a long time. But for clinics, they actually tend to be more flexible. The problem is that we can say that this regulation must require this.

VD2

Table 3 summarizes the 6 major challenges with operational definitions, exemplar quotes, and associated actors (patients, clinicians, midwives, vendors, managers, regulators). We include a concise map here for readability; the expanded map with all subthemes and additional examples appears in Multimedia Appendix 3.

**Table 3:** Major challenges to implementing a maternal-care health recommender system (HRS) with operational definitions, exemplar quotes, and primary actors.

Major challenge and indicator			Operational definition	Exemplar quote	Primary actors
**People**					
	**Skill**				
		Individual factor	Counts when users lack the skill/confidence to navigate the HRS, find and understand needed information, or enter data so they are shifting to paper or other workarounds; excludes technical/device failures.	“Sometimes it is even more complete what is written, which is in a book than what is typed.” [DR2]	BD, DR, GO, PS
**Process**					
	**Accuracy**				
		Incorrect input	Counts when recorded values are factually wrong at entry or later corrupted (manual entry, device miscalibration, app-induced loss); excludes missing/late data.	“Lab system isn’t connected; staff enter results manually; risk of human error.” [DR7]	BD, DR, GO, PS, VD
		Quality of output	Counts when recommendation content or routing is incorrect/ambiguous/misdirected and causing misinterpretation; excludes correct but late (timeliness) or incomplete-input cases.	“Estimated due date in the app was about a month off.” [PS10]	DR, PS, VD
		Partial availability of required data	Counts when only a subset of required inputs is available, preventing accurate computation; excludes wrong values.	“If only patient-reported data are available, recommendations may be inaccurate.” [DR6]	DR
	**Completeness**				
		Lack of data integration	Counts when required data sources (medical records/registers/ devices/government apps) are not linked, causing fragmented records or duplicate entry; excludes wrong values (accuracy) or late arrival (timeliness).	“The issue is it’s not integrated with medical records.” [DR1]	BD, DR, GO, MG, PS
		Partial information	Counts when only a subset of required fields or longitudinal history is available at the point of use, limiting recommendations; excludes integrated but incorrect data.	“They don’t see previous patterns; combining history reveals risk factors.” [VD1]	BD, DR, MG, PS, VD
	**Timeliness**				
		Continuity of maternal care	Counts when ANC service continuity is interrupted because the app/system is unavailable/unstable or lacks timely follow-up mechanisms; excludes accuracy/completeness issues.	“Online apps help initially, but there’s no follow-up with the same doctor.” [DR5]	BD, GO
		Delivery time of service in maternal care	Counts when ANC services, alerts, or information are delivered after recommended clinical windows or too late to act; excludes misrouted/incorrect content (accuracy) or missing inputs (completeness).	“If they must open the app each time, they might miss it.” [VD1]	BD, DR, GO, PS, VD
		Time efficiency in patient handling	Counts when HRS-related steps cause avoidable delays (eg, slow loading, manual entry, multi-screen navigation) that reduce throughput; excludes staffing/scheduling delays unrelated to the HRS.	“There are many patients and limited time so we need to be fast.” [DR10]	DR
**Policy**					
	**Cost**				
		Budget limitation in app implementation	Counts when budget/capacity for development, maintenance, integration, or platform compliance prevents deployment or continuity; excludes standards/regulatory issues (see Standard).	“Access stopped after three years; maintenance and cooperation were constrained.” [GO1]	DR, GO, VD
		Perceived benefit does not justify cost	Counts when stakeholders judge benefits do not justify costs for patients/providers/organizations, reducing willingness to adopt or pay; excludes provider-side budget limits (see row above).	“This technology must be cheap ... anyone should be able to buy it.” [DR4]	PS, DR, GO, VD
	**Standard**				
		Standardization in application development	Counts when lack/inconsistency of shared technical standards or device protocols makes integration or development hard; excludes pure budget issues.	“Because of the custom protocols and no standard, when there’s updates the device integration needs extra efforts.” [VD2]	BD, DR, GO, PS, VD
		Regulatory compliance	Counts when legal or institutional requirements constrain data storage/processing or delay adoption; excludes technical-standard gaps.	“Old law required no data stored on the phone; everything must be sent immediately.” [VD1]	DR, GO, PS, VD

## Discussion

### Principal Findings

The HRS is equipped with the features needed to assist medical professionals in their current roles and the patients they serve [15]. This system provides users with a variety of recommendations that support individuals in taking charge of their health and help medical professionals make the best choices⁠ [15]. This study investigated anticipated challenges and requirements for implementing an HRS for maternity care in Indonesia. We found 6 major challenges regarding skill, accuracy, completeness, timeliness, cost, and standardization, which primarily lie within the people and process dimensions. These 6 major challenges were mentioned 96 times, or 64.43% of all the codes extracted from the interviews. Our analysis of stakeholder interviews suggests a primary focus on real-time, individual-level recommendations, while surveillance applications remain conceptual at this stage.

HRS can be used by both health care professionals and patients, where health care professionals use the system to access supplementary information while patients use the system to receive health-related content that is based on evidence [15]. Additionally, these systems allow patients not only to browse relevant content but also to receive alerts, notifications, risk predictions, and other actionable guidance through the application [32]. Our study shows that one of the major challenges identified is the varying skill levels of users interacting with the HRS. Users, including health care professionals and patients, may struggle with navigating, understanding, and accessing the correct information in the application. This is combined with difficulties in adding or updating new information. If the system is not user-friendly or intuitive, there is a high risk that users may become frustrated or disengage, which would ultimately affect the quality of care and the overall effectiveness of the system. Poor usability in health information systems can lead to negative outcomes such as medical errors, clinician burnout, and patient disengagement [47]. Improving health IT usability is not possible without actively involving users in the design, testing, and implementation stages to ensure the system aligns with their real-world practices and enhances their work rather than creating additional challenges [47]. Priambodo et al [36] discovered that simplicity is a key factor in implementing HRS for maternal care. The app should deliver information quickly, raise awareness, and be user-friendly. Although a significant amount of information needs to be entered, the data recording process should remain simple and clear to ensure that important messages are not missed, enabling both patients and doctors to make informed decisions [48]. Meanwhile, Abejirinde et al [26] found that, despite the perceived usefulness and motivation of users, individual factors such as low technological self-efficacy and limited knowledge hindered the use of the system.

Second, our study shows that accuracy in data entry and processing is a critical concern. Manual input by health care professionals or patients may lead to errors, creating biases or discrepancies in the data. These inaccuracies can arise from technical issues within the application, such as failure to properly record data or rendering previously entered data unreadable. Ensuring that the devices used for data input are properly calibrated is crucial, as this ensures that the system can capture and process data accurately, particularly for sensitive health measurements. Wang and Preininger [49] also found that errors in measurements from various devices can impact the accuracy and reliability of results. Therefore, it is crucial to develop methods that can effectively model and minimize these measurement errors to ensure accurate physiological data analysis. Technical problems during data entry, such as the inability to correct errors and frequent software malfunctions, could lead to further inconsistencies [50]. Additionally, Shiferaw et al [51] identified instances where data were accidentally deleted, memory cards failed, resulting in data loss, and phones became dysfunctional, rendering them unusable when required. Abejirinde et al [52] also found that a malfunction caused incorrect dates to appear in numerous records from one of the facilities, illustrating how system malfunctions can affect the accuracy of health data. Perceived reliability is essential for user satisfaction and the intention to use interactive IT, as it demonstrates the technology’s ability to deliver services consistently, securely, and accurately as promised [53]. These examples highlight the importance of addressing both manual and technical issues to ensure the accuracy, reliability, and overall user trust in health data systems.

Our study also found that the quality of the recommendations generated by the system is just as important as the accuracy of the input. The recommendations must reach the correct person, specifically the individual responsible for taking action based on the health information. Misinterpretations or inaccurate recommendations can lead to confusion, potentially putting patients at risk. This issue is further compounded when patients feel that the system fails to provide adequate information about their maternal health, which undermines the system’s credibility and trustworthiness. Carlisle et al [54] identified usability issues with the app, including the misinterpretation of obstetric definitions within the app’s fields, which aligns with our findings. Furthermore, Lobach [55] reported that ensuring effective nationwide implementation of clinical decision support systems and knowledge management systems requires more than simply delivering the correct information to the right individual. Lobach [55] also found that a deeper understanding of what constitutes the correct information, and when and how it should be delivered, is necessary to expand the reach of clinical decision support beyond isolated, well-established institutions. This underscores the importance of both the quality and timing of recommendations in maintaining system reliability and patient trust.

In some cases, HRS may not have access to all the necessary data required to generate accurate recommendations, which limits their ability to provide comprehensive and actionable suggestions, ultimately affecting the quality of maternal care. Ensuring that all relevant data are captured and integrated is critical for the system’s effectiveness. The data sources for these systems can include user input or information directly obtained from measurement devices [56]. However, even when the suggested diagnoses are generally adequate, the top recommendation’s accuracy can be compromised by ineffective information collection and the system’s inability to capture subtle clinical clues recognized by health care professionals, making the recommendations less useful and more time-consuming [57]. Reengineering workflows to better integrate eHealth solutions is crucial for enhancing efficiency, optimizing task distribution, ensuring patient safety, and improving the completeness and quality of data collected from patients [58].

Third, our study shows that a significant challenge with current HRS is the lack of integration across various data sources. Ideally, these systems should integrate data from multiple platforms, such as medical records and government databases, enabling both health care professionals and patients to access all necessary information through a single application. However, current systems often only support data collection, lacking alerts, reminders, or actionable recommendations due to limited integration, which reduces their overall effectiveness. A typical HRS framework gathers information about a patient’s health status from sources like electronic medical records or electronic health records through secure network protocols to create personal health records [59]. Clinicians found such systems less useful when they were not integrated with other systems, like pharmaceutical databases that provide information on available medications, leading to recommendations that were not helpful when the prescribed medications were unavailable [57].

Another related challenge identified in this preimplementation study of HRS for maternal care is the incomplete information provided by the system. Both patients and health care workers have noted that the information offered is often insufficient to meet their needs. While the system may simplify data recording, users are not receiving the full range of information required to make informed decisions regarding maternal care. Uncertainty in HRS is also associated with potential risks, such as inaccurate predictions due to user preferences not being fully captured, or difficulties in identifying optimal patterns because of incomplete data [32]. Enhancing the system’s capacity to provide comprehensive, detailed data will be essential for improving maternal care outcomes.

Fourth, our study shows that the continuity of maternal care is essential, and disruptions in the HRS can interrupt health care workers’ ability to provide consistent care. If the application is unavailable or disrupted, it hinders the ability of health care providers to follow up with patients and ensure continuous service. To support uninterrupted care, the system needs to be highly reliable and accessible at all times. DeLone and McLean [60] identified reliability, completeness, and accuracy as key metrics for assessing information quality in an information system. Consequently, hospitals must ensure timely operation and delivery of promised services, implement automated processes, and provide all necessary services to meet patients’ needs, thereby offering excellent public service [41].

Timely service delivery is crucial in maternal care, where delays can have significant consequences for both the mother and child. The system must ensure that health care services and recommendations are delivered promptly to address any immediate health concerns, thereby enhancing the overall quality of care. Saha et al [50] found that clinicians faced difficulties using the application offline when it required internet connectivity to manage beneficiaries’ details. Shiferaw et al [51] also found that while the system generally operated well, disruptions may occur due to poor network connectivity or power outages. Ensuring reliable connectivity was challenging during implementation in low- and middle-income countries [61].

Another benefit of improving the timeliness of the HRS is the potential for greater time efficiency in patient handling. With faster and more accurate data processing, health care providers could manage more patients within the same time frame, leading to improved health care efficiency and the ability to serve a larger population. These systems are designed to reduce the time and effort involved in the health care decision-making process, thereby lowering the overall costs [32].

Fifth, our study shows that the cost of developing and implementing HRS in health care facilities is a major concern. Developing these systems requires specialized resources, and many health facilities face budgetary limitations that prevent them from implementing comprehensive and functional solutions. This financial barrier can slow the adoption of such technologies, particularly in resource-constrained settings.

There is also a concern about the perceived cost-benefit ratio of using the system. Some users, particularly patients, feel that the financial burden of adopting the system does not justify the benefits they receive from it. If the system is seen as expensive but ineffective or difficult to use, this perception will act as a deterrent to widespread adoption. Ensuring that the benefits clearly outweigh the costs will be essential for driving the success of the system.

Sixth, our study shows that a lack of standardization in the development of HRS poses a significant challenge. Without standardized guidelines, it is difficult to ensure that these applications are developed in a way that makes them user-friendly, reliable, and compatible across different platforms. Developing and enforcing standards will streamline the development process, ensuring that applications meet the necessary quality and usability benchmarks. For the long-term viability of scaling up, it is vital to develop standards for the mobile health ecosystem that account for service integration and interoperability [51].

Regulatory compliance is another important challenge. HRS must adhere to government regulations and medical practice standards, ensuring that they align with the legal requirements for health care applications. Health facilities and application developers are obligated to follow these regulations, and failure to comply could result in legal issues, reduced system credibility, and potential harm to patients. Therefore, compliance with regulatory standards is a critical consideration for successful implementation. Adhering to standards ensures that health care services are delivered consistently and meet patients’ needs and expectations [62]. Among various dimensions, the policy dimension is the most crucial and should be clearly defined first to ensure that all hospital activities comply with standards related to personnel, processes, and infrastructure. Without a well-defined and standardized policy, the dimensions of human resources and processes cannot function effectively. To enhance patient trust and offer affordable services, the policy dimension should also include guarantees aligned with fairness principles, as well as compensation or warranties for patients who encounter issues.

The findings of this formative, preimplementation study have significant implications for the implementation of HRS in maternal care. Practically, improving the user interface is essential, as some users struggle to navigate and update information; a simpler, more intuitive design will help both patients and health care providers access and input data more easily. Ensuring that devices used for data input are properly calibrated is also crucial for collecting accurate health data, which is vital for generating reliable, actionable recommendations. Additionally, the system should be reliable and accessible at all times to support continuous care, enabling health care providers to respond to real-time health needs and manage more patients efficiently. At the implementation level, the study underscores the importance of evaluating usability, feasibility, and cost implications, especially in resource-constrained settings, to inform adoption. Furthermore, adherence to regulatory standards and the establishment of standardized guidelines are key to ensuring system quality, safety, and trustworthiness across diverse health care environments.

Consistent with the WHO Global Strategy on Digital Health 2020-2025, these findings highlight the need for interoperable, sustainable, and people-centered digital health systems that strengthen public health surveillance and data-driven maternal care [63]. Similarly, the WHO Classification of Digital Interventions, Services, and Applications in Health (second edition) identifies core digital functions such as client’s health tracking, health care provider decision support, and data collection management that align closely with the capabilities of the maternal HRS examined in this study [64].

Although infrastructure challenges were not among the most frequently cited issues in this formative study, participants noted that reliable connectivity, compatible devices, and system interoperability are essential prerequisites for sustained system performance and adoption. These considerations also align with the WHO Global Strategy on Digital Health 2020-2025, where accessibility and interoperability are identified as strategic objectives for developing scalable and equitable digital technologies for global health [63]. Aligning the design and implementation of the maternal HRS with these global frameworks can therefore enhance scalability, interoperability, and equitable integration into national health information systems, ultimately contributing to improved maternal health outcomes.

### Limitations

This study has several limitations that should be considered when interpreting the findings. First, the sample size distribution among stakeholder roles was uneven, with some groups being more represented than others, potentially limiting the diversity of perspectives captured. Additionally, most patients interviewed were from private clinics, with only a few from government-run community health centers, which may lead to findings that primarily reflect experiences typical of private health care settings. Furthermore, all participants lived or worked in the Greater Jakarta area, where IT infrastructure is sufficient, health care facilities are accessible, and quality medical care is affordable. This urban-centric sampling approach may limit the transferability of our findings to rural or resource-limited settings, where differences in health care access and infrastructure could affect how HRS are implemented. We did not design or evaluate aggregation or data feeds for facility- or population-level surveillance; these implications remain conceptual and require future development and testing. Future research should aim for a more balanced sample and include perspectives from rural or underserved areas to provide a broader understanding of these challenges.

Additionally, usability, feasibility, and pilot studies are needed prior to any effectiveness evaluation. Developing an architecture for maternal HRS based on user needs would be valuable for enhancing system relevance and usability. Beyond cross-stakeholder triangulation, future studies should also integrate regulatory documents and cost data to strengthen confirmability and economic interpretation. In this study, we conducted a limited document review (program guidelines and internal standard operating procedure excerpts) to contextualize perceived barriers.

### Conclusions

This formative, preimplementation qualitative study highlights 6 major, stakeholder-perceived challenges to implementing an HRS for maternal care in Indonesia: skills, data accuracy, data completeness, data timeliness, cost/sustainability, and standardization/interoperability. These challenges, primarily within the people and process dimensions, present significant barriers to the successful implementation of the system. Addressing these issues will require a multifaceted approach, including enhancing user training and system usability, ensuring data accuracy and integration, improving the reliability and timeliness of services, managing costs effectively, and establishing clear regulatory standards.

The findings of this research underline the importance of user involvement throughout the system’s design and implementation process to ensure that it meets the needs of both health care professionals and patients. Moreover, technical improvements, such as reducing manual input errors and enhancing system reliability, will be critical for building trust and ensuring that the system supports effective decision-making. Finally, ensuring regulatory compliance and developing standardized guidelines will be essential for facilitating broader implementation and ensuring the system’s long-term success in improving maternal care outcomes in Indonesia.

## Data Availability

The datasets generated or analyzed during this study are available from the corresponding author on reasonable request.

## Appendix

Multimedia Appendix 1 COREQ checklist.

Multimedia Appendix 2 Interview questions.

Multimedia Appendix 3 Summary of challenges.

## Abbreviations

ANC: antenatal care
COREQ: Consolidated Criteria for Reporting Qualitative Research
HEALTHQUAL: Healthcare Service Quality
HRS: health recommender system
SERVQUAL: Service Quality
WHO: World Health Organization

## References

[1] (2019). Maternal mortality: evidence brief. World Health Organization.

[2] (2023). Trends in maternal mortality 2000 to 2020: estimates by WHO, UNICEF, UNFPA, World Bank Group and UNDESA/Population Division. World Health Organization.

[3] Habte A, Tamene A, Melis T (2024). Compliance towards WHO recommendations on antenatal care for a positive pregnancy experience: timeliness and adequacy of antenatal care visit in Sub-Saharan African countries: evidence from the most recent standard demographic health survey data. PLoS One.

[4] Say L, Chou D, Gemmill A, Tunçalp Ö, Moller A, Daniels J, Gülmezoglu AM, Temmerman M, Alkema L (2014). Global causes of maternal death: a WHO systematic analysis. Lancet Glob Health.

[5] Griffin KM, Oxford-Horrey C, Bourjeily G (2022). Obstetric disorders and critical illness. Clin Chest Med.

[6] (2024). Maternal mortality fact sheet no. 348. World Health Organization.

[7] (2016). WHO recommendations on antenatal care for a positive pregnancy experience. World Health Organization.

[8] Sinkey RG, Battarbee AN, Bello NA, Ives CW, Oparil S, Tita ATN (2020). Prevention, diagnosis, and management of hypertensive disorders of pregnancy: a comparison of international guidelines. Curr Hypertens Rep.

[9] (2018). WHO recommendations on antenatal care for a positive pregnancy experience: summary: highlights and key messages from the World Health Organization's 2016 global recommendations for routine antenatal care (No. WHO/RHR/18.02). World Health Organization.

[10] Cameron L, Contreras Suarez D, Cornwell K (2019). Understanding the determinants of maternal mortality: an observational study using the Indonesian population census. PLoS One.

[11] Syairaji M, Nurdiati DS, Wiratama BS, Prüst ZD, Bloemenkamp KWM, Verschueren KJC (2024). Trends and causes of maternal mortality in Indonesia: a systematic review. BMC Pregnancy Childbirth.

[12] Baharuddin M, Amelia D, Suhowatsky S, Kusuma A, Suhargono MH, Eng B (2019). Maternal death reviews: a retrospective case series of 90 hospital-based maternal deaths in 11 hospitals in Indonesia. Int J Gynaecol Obstet.

[13] Hafez R, Meilissa Y, Izati Y (2023). Indonesia's health labor market: a descriptive analysis. World Bank Group.

[14] Wulandari RD, Laksono AD, Nantabah ZK, Rohmah N, Zuardin Z (2022). Hospital utilization in Indonesia in 2018: do urban-rural disparities exist?. BMC Health Serv Res.

[15] Saha J, Chowdhury C, Biswas S, Dash S, Acharya BR, Mittal M, Abraham A, Kelemen A (2020). Review of machine learning and deep learning based recommender systems for health informatics. Deep Learning Techniques for Biomedical and Health Informatics.

[16] Pustozerov E, Popova P, Tkachuk A, Bolotko Y, Yuldashev Z, Grineva E (2018). Development and evaluation of a mobile personalized blood glucose prediction system for patients with gestational diabetes mellitus. JMIR Mhealth Uhealth.

[17] Abejirinde IO, Douwes R, Bardají A, Abugnaba-Abanga R, Zweekhorst M, van Roosmalen J, De Brouwere V (2018). Pregnant women's experiences with an integrated diagnostic and decision support device for antenatal care in Ghana. BMC Pregnancy Childbirth.

[18] Palmer KR, Tanner M, Davies-Tuck M, Rindt A, Papacostas K, Giles ML, Brown K, Diamandis H, Fradkin R, Stewart AE, Rolnik DL, Stripp A, Wallace EM, Mol BW, Hodges RJ (2021). Widespread implementation of a low-cost telehealth service in the delivery of antenatal care during the COVID-19 pandemic: an interrupted time-series analysis. Lancet.

[19] Venkateswaran M, Ghanem B, Abbas E, Khader KA, Ward IA, Awwad T, Baniode M, Frost MJ, Hijaz T, Isbeih M, Mørkrid K, Rose CJ, Frøen JF (2022). A digital health registry with clinical decision support for improving quality of antenatal care in Palestine (eRegQual): a pragmatic, cluster-randomised, controlled, superiority trial. Lancet Digit Health.

[20] Cochran G, Smid MC, Krans EE, Bryan MA, Gordon AJ, Lundahl B, Silipigni J, Haaland B, Tarter R (2019). A pilot multisite study of patient navigation for pregnant women with opioid use disorder. Contemp Clin Trials.

[21] Peleg M, Shahar Y, Quaglini S, Fux A, García-Sáez G, Goldstein A, Hernando ME, Klimov D, Martínez-Sarriegui I, Napolitano C, Parimbelli E, Rigla M, Sacchi L, Shalom E, Soffer P (2017). MobiGuide: a personalized and patient-centric decision-support system and its evaluation in the atrial fibrillation and gestational diabetes domains. User Model User-Adap Inter.

[22] Humphries B, León-García M, Bates S, Guyatt G, Eckman M, D'Souza R, Shehata N, Jack S, Alonso-Coello P, Xie F (2021). Decision analysis in SHared decision making for thromboprophylaxis during pregnancy (DASH-TOP): a sequential explanatory mixed methods pilot study protocol. BMJ Open.

[23] Rahman A, Friberg IK, Dolphyne A, Fjeldheim I, Khatun F, O'Donnell B, Pervin J, Rahman M, Rahman AMQ, Nu UT, Sarker BK, Venkateswaran M, Frøen JF (2021). An electronic registry for improving the quality of antenatal care in rural Bangladesh (eRegMat): protocol for a cluster randomized controlled trial. JMIR Res Protoc.

[24] Akbulut A, Ertugrul E, Topcu V (2018). Fetal health status prediction based on maternal clinical history using machine learning techniques. Comput Methods Programs Biomed.

[25] Simbolon O, Widyawati MN, Kurnianingsih K, Kubota N, Ng N (2020). Predicting the risk of preeclampsia using soft voting-based ensemble and its recommendation.

[26] Abejirinde IO, Zweekhorst M, Bardají A, Abugnaba-Abanga R, Apentibadek N, De Brouwere V, van Roosmalen J, Marchal B (2018). Unveiling the black box of diagnostic and clinical decision support systems for antenatal care: realist evaluation. JMIR Mhealth Uhealth.

[27] Perry MF, Coyote EI, Austad K, Rohloff P (2021). Why women choose to to seek facility-level obstetrical care in rural Guatemala: a qualitative study. Midwifery.

[28] Nsugbe E, Obajemu O, Samuel OW, Sanusi I (2021). Enhancing care strategies for preterm pregnancies by using a prediction machine to aid clinical care decisions. Mach Learn Appl.

[29] Lindahl C, Wagner S, Uldbjerg N, Schlütter JM, Bertelsen O, Sandager P (2019). Effects of context-aware patient guidance on blood pressure self-measurement adherence levels. Health Informatics J.

[30] Etemadi M, Bazzaz Abkenar S, Ahmadzadeh A, Haghi Kashani M, Asghari P, Akbari M, Mahdipour E (2023). A systematic review of healthcare recommender systems: open issues, challenges, and techniques. Expert Syst Appl.

[31] De Croon R, Van Houdt L, Htun NN, Štiglic G, Vanden Abeele V, Verbert K (2021). Health recommender systems: systematic review. J Med Internet Res.

[32] Tran TNT, Felfernig A, Trattner C, Holzinger A (2020). Recommender systems in the healthcare domain: state-of-the-art and research issues. J Intell Inf Syst.

[33] Pincay J, Teran L, Portmann E (2019). Health recommender systems: a state-of-the-art review.

[34] Peleg M, Shahar Y, Quaglini S, Broens T, Budasu R, Fung N, Fux A, García-Sáez G, Goldstein A, González-Ferrer A, Hermens H, Hernando ME, Jones V, Klebanov G, Klimov D, Knoppel D, Larburu N, Marcos C, Martínez-Sarriegui I, Napolitano C, Pallàs À, Palomares A, Parimbelli E, Pons B, Rigla M, Sacchi L, Shalom E, Soffer P, van Schooten B (2017). Assessment of a personalized and distributed patient guidance system. Int J Med Inform.

[35] Pustozerov EA, Chernykh VY, Popova PV, Vasyukova EA, Tkachuk AS, Yuldashev ZM (2020). Health monitoring system for patients with gestational diabetes mellitus based on nutrition diaries and fitness bracelets. Biomed Eng.

[36] Priambodo R, Handayani PW, Sensuse DI, Suryono RR (2022). Health recommender system for maternal care implementation challenges: a qualitative analysis of physicians' perspective.

[37] Tas PG, Sun G, Rezaei J, Brunelli M, Mohammadi M (2023). Emergency service quality assessment using SERVQUAL and BWM. Advances in Best-Worst Method.

[38] Gilavand A, Torabipour A (2022). The quality of services of Iran university hospitals based on SERVQUAL's evaluation model: a systematic review and meta-analysis. Front Public Health.

[39] Lee D (2016). HEALTHQUAL: a multi-item scale for assessing healthcare service quality. Serv Bus.

[40] Nemati R, Bahreini M, Pouladi S, Mirzaei K, Mehboodi F (2020). Hospital service quality based on HEALTHQUAL model and trusting nurses at Iranian university and non-university hospitals: a comparative study. BMC Nurs.

[41] Handayani PW, Hidayanto AN, Sandhyaduhita PI, Ayuningtyas D, Kasiyah (2015). Strategic hospital services quality analysis in Indonesia. Expert Syst Appl.

[42] Kerasidou A, Bærøe K, Berger Z, Caruso Brown AE (2020). The need for empathetic healthcare systems. J Med Ethics.

[43] de la Perrelle L, Radisic G, Cations M, Kaambwa B, Barbery G, Laver K (2020). Costs and economic evaluations of quality improvement collaboratives in healthcare: a systematic review. BMC Health Serv Res.

[44] Tong A, Sainsbury P, Craig J (2007). Consolidated criteria for reporting qualitative research (COREQ): a 32-item checklist for interviews and focus groups. Int J Qual Health Care.

[45] Harahap NC, Handayani PW, Hidayanto AN (2022). Barriers and facilitators of personal health record adoption in Indonesia: health facilities' perspectives. Int J Med Inform.

[46] O’Connor C, Joffe H (2020). Intercoder reliability in qualitative research: debates and practical guidelines. Int J Qual Methods.

[47] Carayon P, Hoonakker P (2019). Human factors and usability for health information technology: old and new challenges. Yearb Med Inform.

[48] Shah AD, Quinn NJ, Chaudhry A, Sullivan R, Costello J, O'Riordan D, Hoogewerf J, Orton M, Foley L, Feger H, Williams JG (2019). Recording problems and diagnoses in clinical care: developing guidance for healthcare professionals and system designers. BMJ Health Care Inform.

[49] Wang F, Preininger A (2019). AI in health: state of the art, challenges, and future directions. Yearb Med Inform.

[50] Saha S, Quazi ZS (2022). Does digitally enabling frontline health workers improve coverage and quality of maternal and child health services? Findings from a mixed methods evaluation of TECHO+ in Gujarat. Front Public Health.

[51] Shiferaw S, Workneh A, Yirgu R, Dinant G, Spigt M (2018). Designing mHealth for maternity services in primary health facilities in a low-income setting - lessons from a partially successful implementation. BMC Med Inform Decis Mak.

[52] Abejirinde IO, De Brouwere V, van Roosmalen J, van der Heiden M, Apentibadek N, Bardají A, Zweekhorst M (2019). Viability of diagnostic decision support for antenatal care in rural settings: findings from the Bliss4Midwives intervention in Northern Ghana. J Glob Health.

[53] Alam MZ, Hoque MR, Hu W, Barua Z (2020). Factors influencing the adoption of mHealth services in a developing country: a patient-centric study. Int J Inf Manage.

[54] Carlisle N, Watson HA, Seed PT, Carter J, Kuhrt K, Tribe RM, Shennan AH (2021). Impact of a medical mobile phone app (QUiPP) for predicting preterm birth on the anxiety and decisional conflicts faced by women in threatened preterm labour. Midwifery.

[55] Lobach DF, Berner ES (2016). Evaluation of clinical decision support. Clinical decision support systems: Theory and practice.

[56] Priambodo R, Wuri Handayani P, Fathoni Aji R (2023). Maternal recommender system systematic literature review: state of the art and future studies. IJIKM.

[57] Wang D, Wang L, Zhang Z, Wang D, Zhu H, Gao Y, Fan X, Tian F (2021). 'Brilliant AI Doctor' in rural clinics: challenges in AI-powered clinical decision support system deployment.

[58] Granja C, Janssen W, Johansen MA (2018). Factors determining the success and failure of eHealth interventions: systematic review of the literature. J Med Internet Res.

[59] Rana P, Jain N, Mittal U, Sharma S, Singh H (2020). An introduction to basic concepts on recommender systems. Recommender System with Machine Learning and Artificial Intelligence Wiley.

[60] DeLone WH, McLean ER (2016). Information systems success measurement. Found Trends Inf Syst.

[61] Labrique AB, Wadhwani C, Williams KA, Lamptey P, Hesp C, Luk R, Aerts A (2018). Best practices in scaling digital health in low and middle income countries. Global Health.

[62] Mosadeghrad AM (2013). Healthcare service quality: towards a broad definition. Int J Health Care Qual Assur.

[63] World Health Organization (2021). Global Strategy on Digital Health 2020-2025.

[64] World Health Organization (2023). Classification of Digital interventions, Services and Applications in Health: A Shared Language to Describe the Uses of Digital Technology for Health.

