# Supplementary material for: Challenges for a Maternal-Care Health Recommender System in Indonesia: Formative Preimplementation Qualitative Study
Source: JMIR Form Res. 2026 Feb 6;10:e73726. doi: 10.2196/73726 (PMC12924044; doi:10.2196/73726)
Supplement: Multimedia Appendix 2 [file formative_v10i1e73726_app2.docx]

## Multimedia Appendix 1 Interview Questions

| Health Workers | Regulator | Application Vendor | Patients | Health Facility Management |
| --- | --- | --- | --- | --- |
|  |  |  |  |  |
| **People**   - What is needed to ensure user satisfaction with the information system for maternal care or existing health applications - How important is access to maternal health information for patients and health workers - What are the obstacles or challenges you face in using information systems for maternal care or other health applications   **Process**   - What functional needs or features need to be implemented in the recommender system for maternal care - Will the use of maternal care applications make it easier to monitor and predict risks - How is the integration or interoperability of health applications owned by hospitals or clinics with existing maternal care applications - Are there concerns about the misuse of data used in health applications  1. Are there any uncertainties regarding the accuracy of health data in the app   **Infrastructure**   - How to implement an information system for maternal care in the health facility where you work - What are the obstacles or challenges you face in using information systems for maternal care or other health applications - Is there a special unit to manage the information system for maternal care in health facilities - Do you receive training to use the maternal care application   **Policy**   - What is the role of health facilities in supporting the implementation and use of information systems for maternal care or existing health applications - Are there any regulations or policies of healthcare facilities that support the implementation of the system - Are there any international regulations or policies that serve as a reference in implementing a recommender system for maternal care or similar applications - Is there encouragement from health facilities or other stakeholders for the use of the recommender system for maternal care in Indonesia? Is there any encouragement from outside parties to adopt a recommender system for maternal care? - Are there regulations or policies from the Indonesian Ministry of Health/IDI that support the implementation of the system - Are there any national and international regulations or policies that can support the implementation of the recommender system for maternal care in health facilities? - What is the role of the Indonesian Ministry of Health/IDI in supporting the implementation of the recommender system for maternal care in Indonesia | **People**   - What is needed to ensure public satisfaction with applications related to maternal care or existing health applications - How important is access to maternal health information for patients and health workers - What are the obstacles or challenges faced in the implementation and use of information systems for maternal care or other health applications?   **Process**   - What functional needs or features need to be implemented in the recommender system for maternal care - Will the use of maternal care applications make it easier to monitor and predict risks - What is the role of the Indonesian Ministry of Health/BPJS Kesehatan to support integration between health applications in Indonesia? How important is the integration between health applications for the Indonesian Ministry of Health/BPJS Kesehatan   **Infrastructure**   - Which health facilities have implemented applications related to maternal care? What features are in the application - What are the obstacles or challenges faced in the implementation of applications related to maternal care or other health applications - Is there a special unit at the Ministry of Health that functions to manage the development of recommender systems for maternal care or other health applications - What health information systems or applications have been developed by the Indonesian Ministry of Health currently? Who is the user of the application   **Policy**   - What is the role of the Indonesian Ministry of Health/BPJS Kesehatan in supporting the implementation of the recommender system for maternal care in health facilities in Indonesia? Are there regulations or policies from the Ministry of Health that support the implementation of the system - Does the Ministry of Health also develop a recommender system application for maternal care or an application that can be used by patients to access maternal health information in health facilities - Are there any international regulations or policies that are a reference for the Ministry of Health in implementing a recommender system for maternal care or other health applications - Is there encouragement from health facilities or other stakeholders to implement a recommender system for maternal care in Indonesia? - Is there any financial support provided by the Ministry of Health to implement a recommender system for maternal care in health facilities - What is the relationship between the Ministry of Health and other stakeholders in the implementation of the recommender system for maternal care or other health applications | **People**   - In your opinion, what characteristics must be met by health applications for maternal care in order to be widely accepted - How does your organization ensure the usability or ease of use of health applications - What are the obstacles or challenges faced in the implementation of health applications for maternal care today? What efforts can be made to overcome these obstacles or challenges   **Process**   - What functional needs or features need to be implemented in the future - What data is exchanged between the applications? What is the concept and mechanism of integration or data exchange between these health applications. What are the benefits and risks of exchanging data with other stakeholders - How to integrate health applications with other stakeholders? What applications are integrated with health applications developed by your organization? - How does your organization ensure data security and privacy on health applications? - How your organization ensures the quality of data on health applications - What medical resume data related to maternal care can patients access on the health application?   **Infrastructure**   - What functional needs or features need to be implemented in the future - What are the obstacles or challenges faced in the implementation of health applications for maternal care today? What efforts can be made to overcome these obstacles or challenges   **Policy**   - What is the role of the Indonesian Ministry of Health in supporting the implementation of the recommender system for maternal care in health facilities in Indonesia? Are there regulations or policies from the Ministry of Health that support the implementation of the system - What national regulations are your organization's reference in the development of health applications? - What international regulations or standards are your organization's reference in the development of health applications? | **People**   - What is needed to ensure the satisfaction of users/pregnant women with the information system for maternal care or existing health applications? - How important is access to maternal health information for patients/pregnant women - What are the obstacles or challenges faced by mothers in using information systems for maternal care or other health applications   **Process**   - What functional needs or features need to be implemented in the recommender system for maternal care - Will the use of maternal care applications make it easier to monitor and predict risks - How is the integration or interoperability of health applications owned by hospitals or clinics with existing maternal care applications  1. Are there concerns about the misuse of data used in health applications 2. Are there any uncertainties regarding the accuracy of health data in the app   **Infrastructure**   - How to use the information system for maternal care in health facilities where mothers check their pregnancies - What are the obstacles or challenges faced by mothers in using information systems for maternal care or other health applications - Did you get training to use the maternal care app   **Policy**   - What is the role of health facilities in supporting the use of information systems for maternal care or existing health applications? Whether there are any regulations or policies of healthcare facilities that support the use of the system - What is the role of the Indonesian Ministry of Health/IDI in supporting the use of the recommender system for maternal care in Indonesia? Do you know that there are regulations or policies from the Ministry of Health/IDI/international institutions that support the use of the system? - Is there encouragement from health facilities or other parties for the use of recommender systems for maternal care | **People**   - What is needed to ensure user satisfaction with the information system for maternal care or existing health applications - How important is access to maternal health information for patients and health workers - What are the obstacles or challenges faced in the implementation and use of information systems for maternal care or other health applications   **Process**   - What functional needs or features need to be implemented in the recommender system for maternal care - Will the use of maternal care applications make it easier to monitor and predict risks - How is the integration or interoperability of health applications owned by hospitals or clinics with existing maternal care applications? What is the mechanism for integrating information systems for maternal care with other applications? Can the system be integrated with other health facilities - Are there concerns about the misuse of data used in health applications - Are there any uncertainties regarding the accuracy of health data in the app - How is the business process of managing information systems for maternal care in health facilities (how the information system is used)   **Infrastructure**   - What is the information system for maternal care in this health facility? What health information systems or applications are currently used by health facilities - What are the obstacles or challenges faced in the implementation and use of information systems for maternal care or other health applications - Is there a special unit to manage the information system for maternal care in health facilities   **Policy**   - What is the role of health facilities in supporting the implementation and use of information systems for maternal care or existing health applications - Whether there are regulations or policies that support the implementation of the system - Are there any national and international regulations or policies that can support the implementation of information systems or recommender systems for maternal care in health facilities? - Is there an information system or application that patients can use to access information or personal data in the health facility - How is the financial support to implement the information system for maternal care or existing health applications - What is the main motivation of health facilities to use information systems for maternal care. |
