# Supplementary material for: Challenges for a Maternal-Care Health Recommender System in Indonesia: Formative Preimplementation Qualitative Study
Source: JMIR Form Res. 2026 Feb 6;10:e73726. doi: 10.2196/73726 (PMC12924044; doi:10.2196/73726)
Supplement: Multimedia Appendix 3 [file formative_v10i1e73726_app3.docx]

## Multimedia Appendix 2 Summary of Challenges

| Dimension | Theme | Sub-theme | Challenge | Challenge Description | Respondents |
| --- | --- | --- | --- | --- | --- |
|  |  |  |  |  |  |
| **People** | Empathy | Personalization | Need of individualized and specific maternal information | Pregnant women need health information that specific in maternal and related to their personal condition | DR1, DR10, PS1, PS10, PS15, PS8 |
|  |  | Communication | Interactive communication | Patient need interactive communication to get immediate feedback about their condition and health worker need to follow up patient's condition.  Patient feels unnecessary to use an app because access to health worker is easy | BD2, DR12, DR3, DR9, PS1, PS15, GO3 |
|  | Professionalism | Innovation | Time and effort in new tech delivery | Delivering new technology takes time to discuss and not easy to find product market fit or product solution fit  Efforts taken in implementation from introducing the new system, training physicians, and running parallel before fully used | GO2, VD2, DR4, DR8, GO2, GO4 |
|  |  | Skill | Individual factor | People using the app should not face difficulties navigating, understanding, accessing the correct information, or when adding new information. | BD2, BD3, DR1, DR10, DR12, DR2, DR4, DR5, DR6, DR8, DR9, GO4, PS1, PS14, PS15, PS5, PS6, PS8, PS9 |
| **Process** | Reliability | Accuracy | Incorrect input | Manual input may result in inaccurate data, biases, discrepancies, or data being mixed up.  Input errors can occur due to technical issues with the application, either preventing data from being entered properly or causing previously inputted data to become unreadable later.  Calibrating the devices ensures that they will be sufficiently sensitive for accurate measurements | BD1, DR7, GO3, PS1, VD1, GO4, DR11, DR2, DR5, DR6 |
|  |  |  | Quality of output | Recommendations must be delivered to the right person, specifically the one responsible for taking action based on it.  Misinterpretation or inaccurate recommendation must be avoided to prevent confusion  Patients assume the app does not provide the required information about her maternal condition. | DR5, DR2, DR3, DR4, DR5, PS10, PS13  DR12, DR8, PS1, PS12, VD2 |
|  |  |  | Partial availability of required data | Recommendations cannot be accurate if only some of the required data is available. | DR6, DR8 |
|  |  | Integrity | Data integrity and system reliability | Clear accountability for who enters and manages the data is essential, especially given concerns about data safety.  Frequent system errors or network problems potentially compromising the reliability and security of the data. | BD2, DR1, DR3, PS6, PS14, PS7, VD2, GO4 |
|  | Responsiveness | Automatic | Need of automated process | Process should be automatically executed based on input data | DR1, DR5, PS15 |
|  |  |  | Repetitive action by user | Users must repeatedly perform the same process, such as entering the same data into the system. | BD2, DR11, DR2, DR5, GO2, MG1, PS1 |
|  |  | Completeness | Lack of data integration | The apps should have been integrated so that all required information can be accessed in a single application.  The apps currently in use can't generate recommendations, alerts, or even reminders because they aren't integrated with medical records, and their integration with government apps is limited to data collection. | DR1, DR11, BD2, BD3, DR1, DR3, DR8, GO3, GO4, MG1, PS15, PS3, PS4, PS5 |
|  |  |  | Partial information | Information required by patients or health workers are partially provided  Users will get benefit from the app's ability to simplify data recording | BD3, DR7, DR8, DR9, MG1, PS15, PS8, VD1, PS1 |
|  |  | Timeliness | Continuity of maternal care | Health worker's continuous work is interrupted when application is disrupted or not available  System should facilitate follow ups to support continuity of service for the patients | BD1, BD2, BD3, GO1, GO3, DR5 |
|  |  |  | Delivery time of service in maternal care | Needed service in maternal care should be delivered on time | BD2, DR4, DR5, DR6, DR7, DR8, GO1, GO4, PS1, PS6, VD1 |
|  |  |  | Time efficiency in patient handling | More patients can be handled if time efficiency is improved | DR10 |
| **Infrastructure** | Tangible | Equipment | Availability of required equipment & infrastructure | Significant gap in network quality and availability between areas | DR1, DR4, GO4, VD1 |
|  |  |  | Lack of technical support | Technical support is not easy to reach | BD2 |
| **Policy** | Assurance | Compensation | Absence of alternatives for patient monitoring | Inaccessibility of apps and unreliable internet that frequently occur force reliance on manual records and patient reconfirmation  An offline-first approach is required for reliable patient monitoring and data management due to inconsistent internet access  Government should provide the recommendation system and the professional organization should help | BD3, DR11, BD2, DR3, GO3, VD2, DR11, DR12, DR3, PS8, PS9 |
|  |  | Cost | Budget limitation in app implementation | Need specific resources capable to develop health application  Limited budget for developing application in health facilities | GO2, DR1, DR10, DR11, DR5, GO1, VD1 |
|  |  |  | Inequal benefit compared to cost | The use of the system creates a cost burden for patients  Feeling that costs are not worth the benefits | DR4, DR8, PS2, PS3, PS5, PS8, DR3, GO2, VD2 |
|  |  | Standard | Standardization in application development | If standards are available, it will be easier to develop applications, and they will be more user-friendly | BD2, DR1, DR6, GO4, PS1, PS15, VD2 |
|  |  |  | Regulatory compliance | Obligation for health facilities and application developer to follow government regulations and medical practice standards. | DR1, DR12, DR2, DR4, DR6, DR7, DR8, GO2, GO4, PS15, VD1, VD2 |
